# Ethyl Chlorid as a General Anaesthetic

**Published:** 1915-12

**Authors:** 


**Ethyl Chlorid as a General Anaesthetic.** Jacques Carles and André Charrier, Progres Med., Oct., advise using several C.C. at the beginning, then ½ C. C. every three or four minutes. As contraction of the jaws frequently occurs and the nasal passages may be inadequate, the mouth gag should always be available. The inflammability of ethyl chlorid and the occasional failure of muscular relaxation are disadvantages. For long and delicate abdominal operations, chloroform or ether is advised.

